# Emerging Roles of Eosinophils in Bone

**DOI:** 10.1007/s11914-025-00913-6

**Published:** 2025-04-04

**Authors:** Darja Andreev, Pauline Porschitz

**Affiliations:** Center for Regenerative Therapies Dresden (CRTD), Technische Universität (TU) Dresden, 01307 Dresden, Germany

**Keywords:** Eosinophils, Bone homeostasis, Osteoimmunology, Osteoporosis, Osteoclasts, Osteoblasts

## Abstract

**Purpose of the Review:**

Eosinophils are traditionally known for their role in immune defense against parasites and their involvement in various immunopathologies, including eosinophilic airway diseases, eosinophilic dermatoses, and gastrointestinal disorders. However, recent findings from our group and other leading laboratories have broadened this perspective, revealing that eosinophils also play crucial roles in tissue development, homeostasis, and regeneration. This review aims to highlight the regulatory functions of eosinophils within the bone niche and emphasize the importance of further research into their role in bone biology.

**Recent Findings:**

Growing evidence suggests that eosinophils are key regulators of bone metabolism, extending beyond their established roles in immunity and inflammation. They contribute to bone homeostasis by inhibiting osteoclast differentiation, helping to prevent excessive bone resorption in osteoporosis and inflammatory arthritis. Additionally, eosinophils may promote osteoblast-mediated bone formation, modulate the mesenchymal and hematopoietic stem cell niche, and contribute to the bone microenvironment by affecting vascularization and extracellular matrix composition. However, their impact may vary under pathological conditions. Patients with eosinophilic disorders are often at an increased risk of osteoporosis and fragility fractures, though this is largely attributed to disease-related treatments rather than eosinophil activity itself.

**Summary:**

Despite emerging insights into the role of eosinophils in bone biology, the underlying mechanisms remain incompletely understood. Further research is essential to elucidate how eosinophils influence bone physiology and pathology.

## Introduction

Eosinophils develop in the bone marrow, and while they are primarily known to migrate to peripheral tissues, they still constitute approximately 8% of total leukocytes in the bone marrow [1]. Despite their considerable number, research on their role in bone metabolism and interactions with cells of the bone niche remains limited.

Bone is a highly dynamic tissue that undergoes continuous remodeling through the coordinated actions of bone-resorbing osteoclasts and bone-forming osteoblasts. This process is tightly regulated by immune cells and their soluble mediators, a concept known as osteoimmunology [2]. Physiological changes, such as estrogen deficiency, inflammatory diseases, aging, or prolonged immunomodulatory treatments, can significantly disrupt bone metabolism, favoring osteoclast activity and excessive bone resorption over bone formation. This can severely weaken the structure, function, and integrity of bones, increasing their susceptibility to fractures and damage, a pathological process known as osteoporosis [3]. Significant research has focused on the interactions between bone and immune cells, particularly macrophages, neutrophils, and Th1/Th17 cells, which release inflammatory cytokines such as tumor necrosis factor alpha (TNFα), interleukin-1 beta (IL-1β), and IL-6 that drive excessive osteoclast-mediated bone resorption [4]. In contrast, immune cells associated with type 2 immunity, including type 2 innate lymphoid cells (ILC2s) and Th2 cells, have been linked to osteoprotective functions [5]. Eosinophils also fall within this category, yet their specific role in bone metabolism remains largely unexplored.

Given the increasing recognition of eosinophils as key regulators of tissue homeostasis, it is crucial to understand their role within the bone niche. This review aims to summarize current knowledge on eosinophil functions in bone biology, including their interactions with bone-resident cells and their potential impact on bone health and disease. Furthermore, we highlight the need for further research to elucidate the mechanisms by which eosinophils regulate bone remodeling and their potential implications in conditions such as osteoporosis and inflammatory bone loss.

## Biology of Eosinophils

### Development in the Bone Marrow

**Fig. 1:**
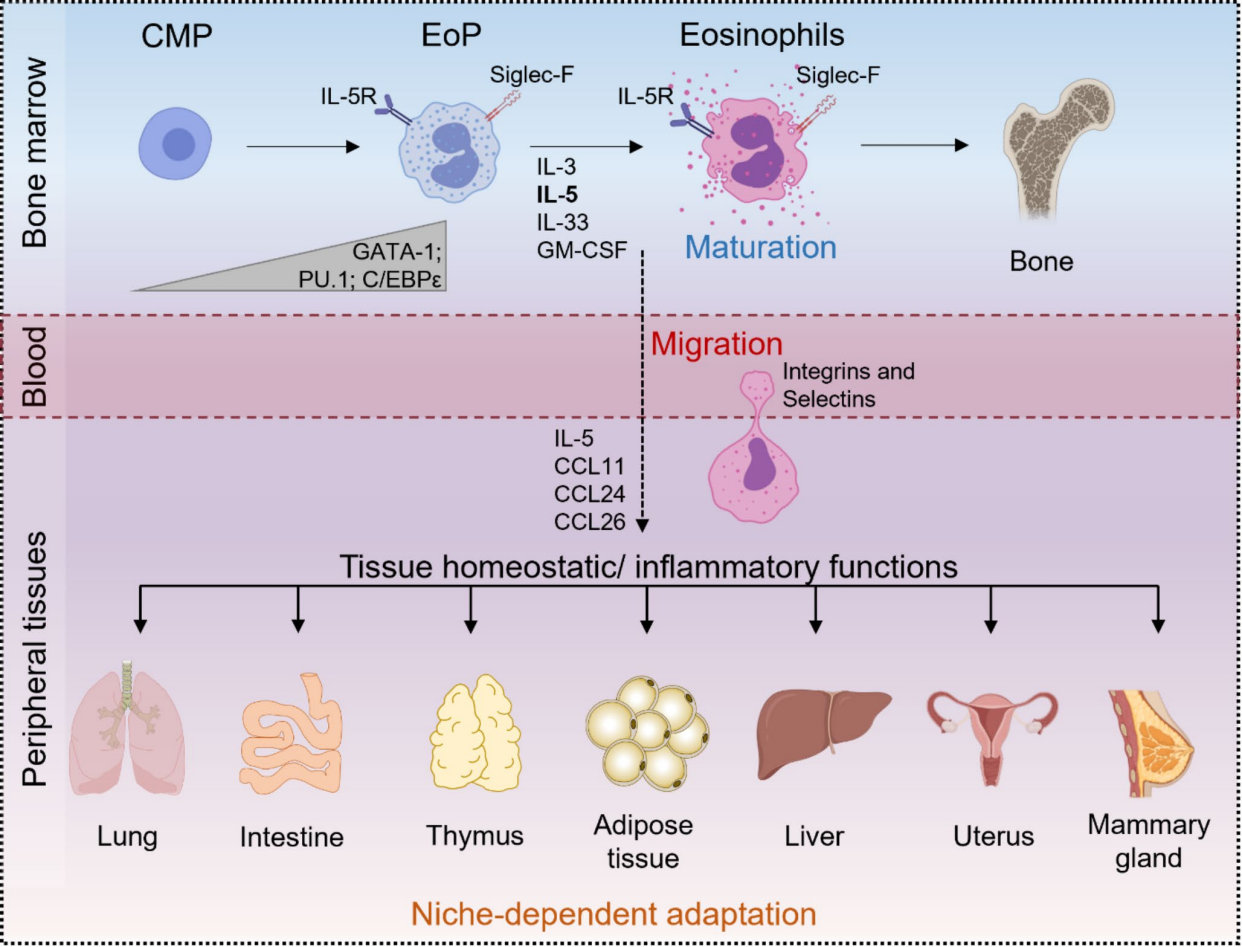
Eosinophil differentiation, migration, and tissue adaptation. Common myeloid progenitors (CMP) in the bone marrow differentiate into eosinophil progenitors (EoP) and subsequently mature into eosinophils under the influence of transcription factors (GATA-1, PU.1, and C/EBPε) and cytokines (IL-3, IL-5 [key cytokine; bold], IL-33, GM-CSF). Eosinophils enter the bloodstream through integrins and selectins and are guided to peripheral tissues via chemotactic signals (IL-5, CCL11, CCL24, CCL26). These cells are predominantly found in tissues characterized by high stem cell activity and ongoing tissue remodeling such as the lung, intestine, thymus, adipose tissue, liver, uterus, mammary gland, and bone. Within these niches, eosinophils adapt to their specific microenvironment, playing roles in either maintaining tissue homeostasis or mediating inflammatory responses

Eosinophils are derived from multipotent hematopoietic stem cells in the bone marrow, which differentiate into eosinophil-committed progenitors (EoPs) in response to cytokines such as interleukin-3 (IL-3), IL-5, IL-33, and granulocyte-macrophage colony-stimulating factor (GM-CSF) [6]. Their differentiation is regulated by key transcription factors including globin transcription factor 1 (GATA-1), PU.1, and members of the CCAAT/enhancer binding protein (C/EBP) family [7]. The essential role of GATA-1 in this process is illustrated by the ΔdblGATA mouse model, which lacks eosinophils but retains other cell lineages due to the specific deletion of the high-affinity GATA-binding site in the GATA-1 promoter [8]. After completing full maturation in the bone marrow, eosinophils enter the circulation and migrate to peripheral tissues following IL-5, eotaxin-1 (C-C motif chemokine ligand 11 [CCL11]), eotaxin-2 (CCL24), and eotaxin-3 (CCL26) chemotaxis [9]. The development and migration of eosinophils to peripheral organs is summarized in Fig. 1.

### Spatial Adaptation

Although eosinophil presence in healthy tissue has long been observed, the dogmatic view of eosinophils as either aggressive effector cells in the innate immune response to parasite infection or drivers of pathophysiological processes in type 2 immune response- associated disorders remains persistent. Only in recent years, the functional significance of tissue-resident eosinophils under homeostatic conditions has been started to be explored. The ***L***ocal ***I***mmunity ***A***nd/or ***R***emodeling/Repair (LIAR) hypothesis of James Lee proposes that eosinophils are, in fact, intrinsically homeostatic cells that actively contribute to the maintenance of tissue homeostasis and exhibit adaptations to the tissue where they reside [10]. The distribution of tissue-resident eosinophils varies widely throughout the body. In most adult homeostatic tissues, they are scarce or entirely absent. For example, in the healthy adult mouse lung, eosinophils constitute only about 1.5% of the total CD45 + hematopoietic cells [11]. Their numbers, however, increase significantly in certain contexts such as in tissues characterized by high turnover rate and stem cell activity including small intestine, uterine lining, bone marrow, and thymus. Moreover, eosinophils are present during developmental processes (e.g., mammary gland ductal differentiation, Peyer’s patch formation, postnatal lung development, beige fat biogenesis), and during normal tissue repair following injury [12]. Elevated eosinophil levels are also observed in diseases involving extensive tissue remodeling, including helminth infections, fibrosis, cancers, and allergic or non-allergic conditions with significant remodeling (e.g., eosinophilic esophagitis, chronic rhinosinusitis) [12]. Notably, the half-life of eosinophils newly released from the bone marrow is estimated to be relatively short, between 3 and 18 h. However, their lifespan can be increased upon migration into peripheral tissues [13]. This effect is accompanied by substantial changes in surface marker expression in dependence on the tissue type, indicating the significance of the tissue microenvironment for eosinophil fine-tuning [14]. Eosinophils harbor a diverse array of bioactive molecules within their granules and cytoplasmic lipid bodies. Their ability to rapidly secrete large quantities of soluble mediators including granule-associated proteins, cytokines, chemokines, growth factors, enzymes, and lipid mediators, places eosinophils as critical regulators of a broad variety of immunological processes [15]. In this context, distinct eosinophil subpopulations with either regulatory or inflammatory properties have been identified in both the lungs and the intestine of rodents and humans [11, 16].

Bone tissue possesses features that assume eosinophil involvement, including active stem cell niches and continuous tissue remodeling. Notably, approximately 10% of calcified bone is remodeled annually in the human body [17]. Eosinophils develop in the bone marrow, and while they are generally thought to migrate to peripheral tissues, the extent to which mature eosinophils are retained within the bone marrow niche, their turnover rate, and their specific functional roles in this environment remain poorly understood. In both mice and humans, eosinophils are estimated to constitute about 8% of all leukocytes in the bone marrow [1]. Given their roles in development, tissue remodeling, and repair, eosinophils are likely contributors to bone homeostasis. Research from our group has begun exploring this hypothesis. Furthermore, insights into eosinophil functions in other tissues may provide valuable clues about their potential roles in bone, though their significance in this context requires further validation.

## Biology of Bone

### Bone Homeostasis

Bones stop growing once adulthood is reached. Nevertheless, the skeleton remains a highly dynamic tissue that undergoes continuous remodeling to maintain skeletal integrity and mineral homeostasis. Bone remodeling is a tightly orchestrated, multi-step process that relies on a delicate balance between the activity of bone-resorbing osteoclasts and bone-forming osteoblasts. The mechanism of bone remodeling consists of four sequential phases: activation, resorption, reversal, and formation [18].

During the activation phase, hematopoietic stem cell-derived osteoclast progenitors are recruited to the site of bone damage, where they undergo cell-cell fusion to form multinucleated bone-resorbing osteoclasts. This process is primarily regulated by the osteoclastogenic cytokines macrophage colony-stimulating factor (M-CSF) and receptor activator of nuclear factor-kappa B ligand (RANKL) [19, 20]. RANKL binds to its receptor RANK on osteoclast precursors, triggering signaling cascades that involve nuclear factor kappa B (NF-κB), mitogen-activated protein kinases (MAPKs), and nuclear factor of activated T cells 1 (NFATc1). These pathways collectively regulate osteoclast differentiation, cytoskeletal organization, and resorptive activity [21]. M-CSF, through its receptor colony-stimulating factor 1 receptor (c-Fms), promotes the survival of osteoclast progenitors while enhancing the expression of RANK and other molecules involved in RANK/NF-κB signaling [22, 23].

The resorption phase follows, during which mature osteoclasts form an adhesive, F-actin-rich sealing zone on the bone surface. This specialized structure enables the targeted release of protons and hydrolases, which dissolve the inorganic bone matrix and expose the organic components. The organic matrix is then degraded by lysosomal proteases, including cathepsin K, matrix metalloproteinases (MMPs), and tartrate-resistant acid phosphatase (TRAP) [4, 24].

Next, during the reversal phase, osteoclasts undergo apoptosis while mesenchymal stem cell (MSC)-derived osteoblast precursors are recruited to the resorption site. A key factor in osteoblast differentiation is the transcription factor runt-related transcription factor 2 (Runx2), which regulates the expression of osteoblast-specific genes such as bone sialoprotein (BSP), osteocalcin (OCN), and alkaline phosphatase (ALP). ALP activity marks the transition of osteoblast progenitors into pro-osteoblasts [25, 26].

Finally, in the formation phase, mature osteoblasts synthesize and deposit new bone matrix components, which are subsequently mineralized [27–29]. Fully differentiated osteoblasts are characterized by an increased osterix (Osx) expression and secrete extracellular proteins, such as collagen type I, BSP I/II, and OCN, which collectively form the organic bone matrix, referred to as osteoid [30–32]. The osteoid is subsequently mineralized by osteoblasts via calcium phosphate accumulation [33]. Osteoblasts typically do not function alone but operate in clusters along the bone surface, lining the bone matrix they produce. Under steady-state conditions, the production and mineralization of bone matrix occur at the same rate, leaving the osteoid completely mineralized [34]. As functional cells, osteoblasts rarely undergo division or proliferation. Instead, 15% of mature osteoblasts are eventually entrapped by the developing bone matrix and gradually transform into osteocytes that reside in the bone matrix and play a key role in mechanotransduction and bone remodeling. Other mature osteoblasts either remain on the bone surface as flat-lining cells or undergo apoptosis [34, 35].

The whole process is completed in approximately 3–6 months [36]. The highly coordinated character of bone remodeling is illustrated by the fact that almost all new bone formation occurs in areas of previous bone resorption, resulting in a constancy of net bone mass and mechanical strength [28, 37].

### Cellular and Molecular Players in Bone Homeostasis and Bone Pathology

**Fig. 2:**
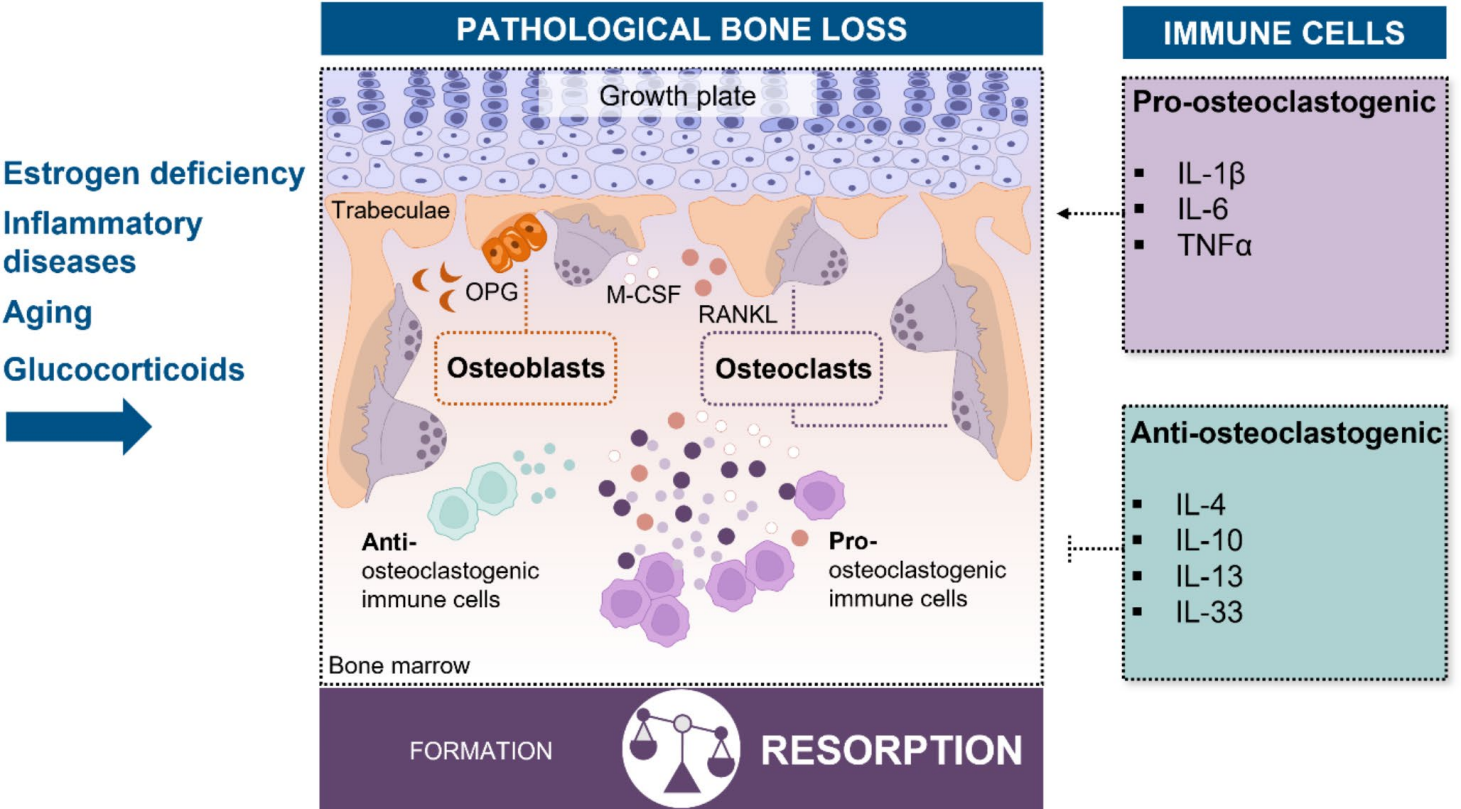
Pathological bone loss and the role of immune cells in osteoclast regulation. Physiological changes, such as estrogen deficiency, inflammatory diseases, aging, and glucocorticoid use, contribute to pathological bone loss by disrupting the balance between bone formation by osteoblasts and resorption by osteoclasts, favoring increased bone resorption. Osteoblasts regulate osteoclast differentiation through the production of macrophage colony-stimulating factor (M-CSF) and receptor activator of nuclear factor kappa-B ligand (RANKL), which promote osteoclast formation and activation. Conversely, they also produce osteoprotegerin (OPG), which inhibits osteoclastogenesis and helps prevent excessive bone resorption. In conditions of bone loss, immune cells, such as macrophages and T cells, release pro-osteoclastogenic cytokines, including IL-1β, IL-6, and TNFα, which enhance osteoclast activity. However, certain immune cells associated with type 2 immune responses secrete cytokines like IL-4, IL-10, IL-13, and IL-33, which suppress osteoclast formation and function, counteracting bone resorption

The homeostasis of bone remains intact as long as the activities of osteoclasts and osteoblasts are in a well-adjusted equilibrium. This balance is shaped by various microenvironmental factors, including cytokines, growth factors, hormones, oxygen levels, nutrition, and mechanical forces. Particularly crucial is the interplay between bone cells and other cells within the bone niche, such as hematopoietic and mesenchymal stem cells, immune cells, adipocytes, and endothelial cells. Consequently, physiological changes, such as estrogen deficiency, inflammatory diseases, aging, or prolonged immunomodulatory treatments, can significantly influence this balance. Disruptions in bone metabolism, particularly those favoring osteoclast activity and excessive bone resorption over bone formation, can severely weaken the structure, function, and integrity of bones, increasing their susceptibility to fractures and damage, a pathological process known as osteoporosis. Figure 2 illustrates the imbalance between bone resorption and formation in osteoporosis and highlights the role of immune cells in regulating osteoclast activity.

Central to the regulation of osteoclasts is RANKL and its decoy receptor osteoprotegerin (OPG), mainly produced by osteoblasts [38]. OPG acts as an inhibitor of RANK-RANKL interplay, thereby preventing osteoclast maturation and activity. The importance of this regulatory network is underscored by the pathological consequences of its disruption. Overexpression of OPG has been shown to result in profound osteopetrosis due to impeded formation of osteoclasts and, ultimately, excessive bone density [39]. In contrast, overexpression of RANKL is associated with severe, early-onset bone loss [40].

RANKL-mediated osteoclastogenesis can be further amplified by inflammatory cytokines such as TNFα, IL-1β, and IL-6. In particular, TNFα and IL-1β interact with their respective receptors on osteoclast precursors, enhancing the M-CSF- and RANKL-mediated signaling cascade [41]. Similarly, IL-6, in conjunction with its soluble receptor (sIL-6R), has been shown to directly promote osteoclast differentiation through interaction with osteoclast precursor cells [42, 43]. Next to directly provoking osteoclast differentiation, TNFα and IL-6 both also induce higher production of RANKL in osteoblasts and stromal cells [44]. Additionally, these cytokines can negatively impact osteoblast function. For example, TNF has been shown to promote the degradation of Runx2 and downregulate the expression of essential bone matrix proteins, including collagen type I, ALP, and OCN, thereby impairing bone formation and mineralization [45, 46]. These inflammatory mediators are primarily secreted by immune cells residing within the bone niche, a concept encapsulated by the field of osteoimmunology [2]. Classical macrophages and T cells are among the key immune cells driving this pro-osteoclastogenic cycle, being particularly prominent in inflammation-mediated bone loss, including rheumatoid arthritis [47].

Conversely, certain immune cells and cytokines exhibit osteoprotective properties. Notably, cytokines associated with type 2 immunity, such as IL-4 and IL-33, have been implicated in counteracting excessive osteoclast-mediated bone resorption [41]. Type 2 immune response-associated cells, including type 2 innate lymphoid cells (ILC2s) and Th2 cells, have been shown to contribute to bone preservation [5, 48]. However, these cells are typically present in low numbers or are absent under homeostatic conditions in bone. A potential alternative source of these cytokines are eosinophils, which develop in the bone marrow and constitute approximately 8% of total white blood cells under homeostasis. Their constant presence within the bone marrow suggests a potential role in regulating bone biology.

### Emerging Evidence of Eosinophil Involvement in Bone

**Fig. 3:**
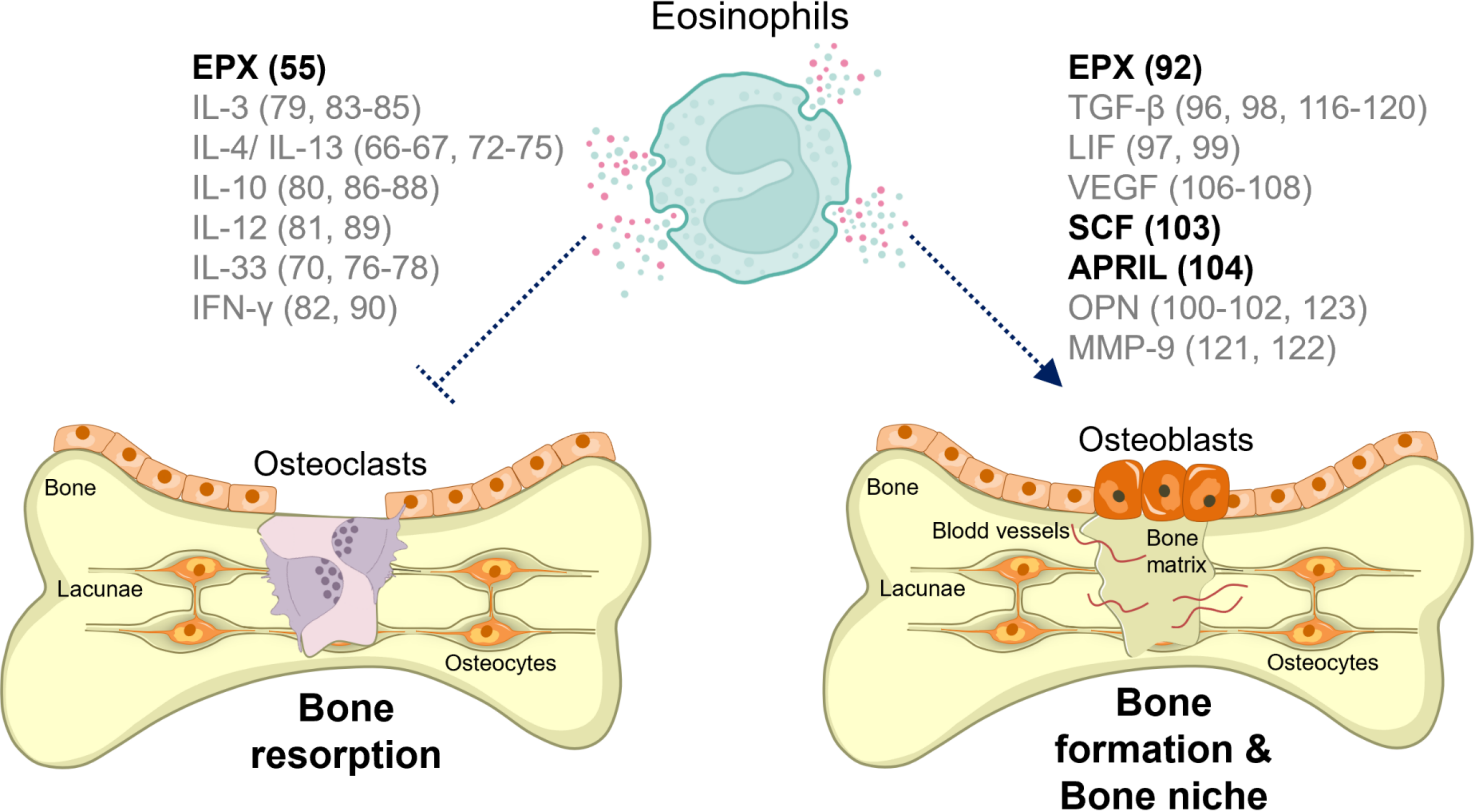
Eosinophils regulate bone homeostasis by influencing bone resorption and potentially also bone formation and the bone niche. They secrete a range of factors that can impact the activity of osteoclasts and osteoblasts. On the left, eosinophils may inhibit bone resorption by releasing factors such as eosinophil peroxidase (EPX), IL-3, IL-4, IL-10, IL-12, IL-13, IL-33, and interferon-gamma (IFN-γ), which modulate osteoclast differentiation and activity. On the right, eosinophils may promote bone formation and influence the bone niche by releasing proteins like EPX, transforming growth factor-beta (TGF-β), leukemia inhibitory factor (LIF), vascular endothelial growth factor (VEGF), stem cell factor (SCF), a proliferation-inducing ligand (APRIL), osteopontin (OPN), and matrix metalloproteinase-9 (MMP-9), which support osteoblast function, bone remodeling, and vascularization. This dual role positions eosinophils as potential regulators of the balance between bone formation and resorption. Proteins directly secreted by eosinophils in the bone marrow are shown in bold, while proteins secreted by eosinophils in other tissues with identified roles in bone biology are highlighted in grey. The relevant references are provided in brackets

Eosinophils originate from hematopoietic stem cells in the bone marrow, and, while most mature eosinophils migrate to peripheral tissues, a subset remains within the bone niche, indicating a potential regulatory role in local immune and remodeling processes. Understanding eosinophil functions in tissues has been greatly advanced through the development of genetically modified mouse models. These include IL-5 transgenic mice, which exhibit eosinophil overexpression [49], and various eosinophil-deficient strains, such as ΔdblGATA (Gata1 deletion) [8], PHIL (*Epx*-driven diphtheria toxin) [50], iPHIL (inducible eosinophil depletion) [51], and EoCre (targeted gene deletion) [52]. Additionally, cytokine reporter mice have provided insights into eosinophil-derived cytokines and their roles in tissue-specific functions [53]. Moreover, recent advances in single-cell transcriptomics have enabled the identification of functionally distinct eosinophil subsets [54]. However, until recently, research on the direct impact of eosinophils on bone niche cells and bone properties, such as density, strength, and regeneration, has been lacking. Figure 3 illustrates the potential role of eosinophils in bone remodeling, emphasizing the confirmed soluble mediators they secrete in the bone marrow, as well as those released by eosinophils in other tissues that are reported to exhibit bone-regulatory properties.

### Impact of Eosinophils on Osteoclasts

Our group was the first to uncover the role of eosinophils in steady-state bone, inflammatory arthritis, and estrogen-loss-induced bone decline in long bones [55]. We found that eosinophil-deficient ΔdblGATA mice exhibited significantly reduced bone mass, whereas hyper-eosinophilic IL-5tg mice showed increased bone mass even under homeostatic conditions. These effects were even more pronounced in disease models, with ΔdblGATA mice experiencing more severe inflammatory arthritis- and estrogen-loss-induced bone decline, while IL-5tg mice were completely protected from pathological bone loss. Mechanistically, eosinophils inhibit bone-resorbing osteoclasts via the release of the heme peroxidase eosinophil peroxidase (EPX). It has been shown in two independent mouse studies that heme peroxidases reduce the level of reactive oxygen species (ROS) in osteoclasts, thereby impairing the RANK-MAPK signaling cascade [55, 56].

When eosinophils are primed by type 2 immune activation, such as during helminth infection or allergic asthma, they accumulate in the joints of arthritic mice, leading to reduced inflammation and diminished subchondral bone erosion [57–59]. This osteoprotective effect may result from a direct anti-osteoclastogenic function of eosinophils or their pro-resolving role in arthritis, where reduced inflammation leads to decreased inflammation-induced bone loss. Interestingly, eosinophils within the synovial tissue exhibited increased secretion of tissue-remodeling factors. In arthritic mice with elevated eosinophil levels, fewer bone lesions and osteophytes were observed once inflammation had fully resolved, suggesting a potential role of eosinophils in bone tissue regeneration [58]. Mechanistically, synovial eosinophils may inhibit inflammatory arthritis through the production of resolvins [58, 60–63] or by promoting the induction of anti-inflammatory macrophages in arthritic joints [57, 64]. The protective effect of *Nippostrongylus brasiliensis* infection against inflammatory arthritis relies on the IL-4/IL-13-induced STAT6 pathway [57], which plays a key role in polarizing anti-inflammatory, alternatively activated macrophages (AAMs) [65]. Eosinophils are a major source of IL-4 and IL-13 [66, 67] and are well known for priming anti-inflammatory macrophages [68, 69]. In line with this, eosinophil deficient ΔdblGATA mice presented reduced numbers of AAMs in arthritic joints [57]. Additionally, eosinophils are able to secrete the alarmin IL-33 [70], which has recently been shown to effectively induce AAMs during muscle regeneration after injury in mice [71].

Notably, IL-4 and IL-33 are not only important for macrophage polarization but also act as potent inhibitors of osteoclast differentiation [44]. IL-4 specifically targets early osteoclast precursors and inhibits RANKL- and TNFα-induced osteoclastogenesis by suppressing the downstream activation of MAPK and NF-κB signaling pathways in vitro [72–74]. The structurally similar IL-13 also binds the IL-4 receptor and amplifies these anti-osteoclastogenic effects [44, 75]. A comparative analysis of the anti-osteoclastogenic effects of IL-4 and IL-13 revealed that IL-4 exerts a more pronounced inhibitory effect on osteoclast differentiation and bone-resorbing activity [74]. Notably, IL-4 and IL-13, to a similar extent, promote the production of anti-osteoclastogenic effector molecule OPG in osteoblasts [75]. In the case of IL-33, it shifts the differentiation balance from osteoclasts to AAMs, thereby inhibiting osteoclast formation and reducing inflammatory bone loss in human TNF-α transgenic mice [76]. IL-33 also has a direct inhibitory effect on osteoclasts by modulating the expression of Blimp-1 and IRF-8 or inducing the apoptosis of osteoclasts in vitro [77, 78].

Other cytokines secreted by eosinophils with anti-osteoclastogenic potential include IL-3, IL-10, IL-12, and interferon-gamma (IFN-γ) [44, 79–82]. IL-3 impairs the differentiation of human osteoclasts and their resorptive capacity by reducing the expression of c-Fms and the osteoclastogenic transcription factors PU.1 and c-Fos [83]. IL-3 inhibits RANKL-induced osteoclastogenesis through signal transducers and activators of transcription 5 (STAT5), as evidenced by STAT5 gain- and loss-of-function mouse models [84]. Moreover, IL-3 is able to downregulate the TNFα-TNFR signaling pathway in osteoclasts in vitro [85]. IL-10 has been reported to inhibit osteoclastogenesis by reducing the expression level and the nuclear translocation of NFATc1 [86]. Additionally, IL-10 is a well-known anti-inflammatory cytokine that suppresses inflammation, thereby helping to mitigate osteoclast-driven bone resorption in different arthritis mouse models [87, 88]. IL-12 inhibits RANKL- and TNFα-induced osteoclastogenesis by promoting cell apoptosis through a Fas/Fas ligand-dependent pathway [89]. This anti-osteoclastogenic mechanism has also been reported for IFN-γ [90].

While the inhibitory role of these mediators on osteoclastogenesis is well established, direct evidence of their secretion by eosinophils within the bone niche remains lacking and requires further investigation. Additionally, it is important to consider that eosinophils, depending on their spatial adaptation, can also produce pro-osteoclastogenic factors such as IL-6, IL-1β, and TNF [9, 91]. Consequently, their overall impact on osteoclasts may vary between homeostasis and disease, as eosinophils could alter their functionality under pathological conditions.

### Impact of Eosinophils on Osteoblasts

The direct influence of eosinophils on osteoblast differentiation from MSCs and their function remains unexplored. However, eosinophil peroxidase has been shown to stimulate collagen I deposition and bone matrix mineralization by osteoblasts in vitro [92]. Additionally, findings by Macias et al. suggest that eosinophils may promote osteoblast differentiation and mineralized bone formation, as hypereosinophilic IL-5tg mice show ectopic bone formation in the spleen and osteopetrosis-like features in long bones [93]. Histological analysis of splenic nodules revealed osteoid matrices, osteocytes within mineralized trabecular plates, and regions of woven and organized lamellar bone. This ossification is unlikely driven directly by IL-5, as osteoblasts and osteoclasts lack the IL-5 receptor [93]. Instead, IL-5tg mice show a dramatic eosinophil increase, comprising 50% of all spleenocytes and 70% of all bone marrow cells, alongside a 30-fold rise in B cells [49]. While B cells are typically linked to bone loss via osteoclast induction in conditions like rheumatoid arthritis and postmenopausal osteoporosis [94, 95], eosinophils secrete cytokines such as transforming growth factor beta (TGF-β) and leukemia inhibitory factor (LIF), which may promote osteoblast differentiation from mesenchymal or osteoprogenitor cells [93, 96–99]. Furthermore, eosinophils inhibit osteoclast activity, potentially contributing to the ectopic bone formation in the spleen and the osteopetrosis-like phenotype in long bones of IL-5tg mice. Despite these associations, further studies are necessary to clarify the role of bone-resident eosinophils in osteoblast formation and mineralized bone deposition.

### Interaction with Cells from the Bone Niche

The bone marrow niche not only harbors bone-specific cells but also serves as a reservoir for stem cells and a key site for immune cell development. Additionally, bone health is intricately regulated by vascularization, and the extracellular matrix (ECM). While direct evidence of eosinophil involvement in these factors within bone remains scarce, insights from other tissues may provide valuable clues applicable to the bone microenvironment.

Research on the role of eosinophils in regulating mesenchymal and hematopoietic stem cells is limited. However, eosinophils are known to produce various growth factors, including vascular endothelial growth factor (VEGF), nerve growth factor (NGF), fibroblast growth factor (FGF), stem cell factor (SCF), TGF-β, and a proliferation-inducing ligand (APRIL) [14]. Eosinophils are an important source of osteopontin (OPN) [100], which has been shown to promote MSCs proliferation and migration [101, 102]. Human peripheral blood eosinophils have been identified as a source of SCF, also known as c-Kit ligand, which plays a crucial role in mast cell differentiation, survival, and activation in the bone marrow [103]. Furthermore, eosinophils contribute to plasma cell maintenance in the bone marrow by producing APRIL and IL-6 in mice [104]. However, ovalbumin-induced airway inflammation leading to eosinophilia has been associated with disrupted hematopoietic stem cell homeostasis through the production of CCL-6 by eosinophils [105].

Vascularization is critical for bone integrity, ensuring an adequate supply of oxygen and nutrients. Eosinophils, as producers of VEGF, are recognized for their pro-angiogenic role in conditions such as asthma [106, 107]. They have been shown to promote new vessel formation in both aortic ring and chick embryo chorioallantoic models [108]. Beyond VEGF, eosinophils secrete additional pro-angiogenic factors, including IL-8, NGF, major basic protein (MBP), and amphiregulin [109–112]. IL-8 facilitates endothelial cell organization into capillary-like tube structures in vitro [113]. NGF enhances angiogenesis in a mouse model of ischemia [114]. MBP, a cationic protein linked to eosinophil activity, has been shown to directly promote angiogenesis both in vitro and in vivo by stimulating capillary formation on Matrigel and inducing sprouting in the CAM assay [111]. Amphiregulin supports endothelial cell tubule formation in fetal mouse lung endothelial-like cells [115]. While eosinophil-driven angiogenesis is often linked to pathological conditions, such as allergic diseases, their regulatory function may also play a beneficial role in maintaining tissue homeostasis and overall health.

The ECM is a complex network of macromolecules that provides structural support to tissues. In bone, the ECM provides both strength and rigidity, ensuring mechanical stability and resilience. Eosinophils have been shown to influence ECM remodeling in both in vitro and in vivo studies, particularly by promoting airway remodeling through myofibroblast differentiation and ECM deposition [12]. This effect is largely attributed to their storage and release of TGF-β, a potent pro-fibrogenic factor [116, 117]. In vitro, eosinophils enhance fibroblast proliferation, ECM synthesis, and lattice contraction, primarily through the secretion of TGF-β and IL-1β [118, 119]. As mentioned earlier, TGF-ß also plays a key role in bone matrix apposition [120]. Additionally, eosinophils store and release MMP-9, a key enzyme involved in ECM remodeling, with eosinophils being a major source of MMP-9 in asthmatic airways [121]. MMPs are also essential for breaking down the organic components of the bone matrix, with MMP-9 playing a particularly important role. Thus, MMP-9 knockout mice display disrupted skeletal growth plate vascularization and ossification, highlighting its essential function in bone development [122]. Moreover, eosinophils secrete the glycoprotein OPN, a component of the bone extracellular matrix that is implicated in maintaining bone homeostasis and contributing to bone pathology [123]. These findings suggest that eosinophils are well-equipped to influence the bone tissue environment. While their role in fibrosis is well-documented, their ability to regulate ECM remodeling may also contribute to normal tissue homeostasis and repair processes in bone.

### Impact of Eosinophils on Bone in Humans

Human studies investigating the impact of eosinophils on bone are limited and primarily focus on the effects of respiratory diseases, such as asthma and chronic obstructive pulmonary disease (COPD), on bone health. Both asthma and COPD patients have an increased risk of osteoporosis and fragility fractures compared to the general population [124]. However, these patients often receive extensive corticosteroid treatment, a known contributor to secondary osteoporosis [125], making it difficult to distinguish the direct effects of elevated eosinophil levels from those of disease treatment on bone. Additionally, osteoporosis associated with these diseases is closely linked to neutrophil-mediated inflammation involving TNFα and IL-6 [126].

Osteoporosis is also a common comorbidity in rheumatoid arthritis (RA) patients [127]. In our recent study we observed a significant correlation between eosinophil counts and eosinophil cationic protein (ECP) levels with trabecular bone mass in both healthy individuals and RA patients [55]. However, in RA patients undergoing glucocorticoid treatment, eosinophil, and ECP levels were generally suppressed, and no relationship with bone mass could be detected. Interestingly, RA patients with concomitant asthma who were in remission for arthritis experienced a relapse of arthritis after treatment with mepolizumab, an IL-5-neutralizing monoclonal antibody that depletes eosinophils [58]. Recent studies have confirmed that biologics targeting the type 2 immune response, particularly omalizumab and mepolizumab used in severe asthma, are associated with an increased incidence of rheumatic diseases [128]. These findings also suggest an inverse correlation between asthma and RA risk, an observation supported by several human cohort studies [129]. However, some studies have reported conflicting results, indicating a higher incidence of arthritis in asthma patients [130]. These discrepancies may arise from the heterogeneity of asthma phenotypes. Asthma can be categorized into neutrophilic and eosinophilic subtypes, each characterized by distinct cytokine profiles [131]. Choy et al. identified three main patient clusters: Th2-high, Th17-high, and Th2/Th17-low asthma, with Th2-high and Th17-high responses being inversely correlated [132]. In a previous study, we could confirm that eosinophilic asthma, associated with a type 2 cytokine signature, promotes the resolution of inflammatory arthritis, whereas IL-17-driven asthma exacerbates arthritis in a murine model [58]. Therefore, future correlation analyses examining the relationship between eosinophil levels and bone in humans should account for the used medication and the disease endotype.

## Conclusion and Future Directions

Although a stable population of eosinophils resides in the bone marrow, studies exploring their role in bone homeostasis and disease are scarce. This lack of research is largely due to the long-standing perception of eosinophils as late-stage effector cells primarily involved in parasite defense and immunopathologies such as atopy. However, advancements in genetic models and technological breakthroughs, including single-cell RNA sequencing, now enable a more detailed characterization of eosinophil populations, providing new opportunities to investigate their tissue-homeostatic functions. In the context of bone, our research is at the forefront of uncovering the role eosinophils play in maintaining skeletal integrity. So far, these cells have been shown to interact with osteoclasts, helping regulate excessive bone resorption. Additionally, emerging evidence suggests that eosinophils may influence osteoblast-mediated bone formation, modulate the mesenchymal and hematopoietic stem cell niche, and contribute to the bone microenvironment, including vascularization and extracellular matrix composition.

In humans, eosinophils are often considered of minor significance due to their relatively low numbers in circulation, especially when compared to neutrophils, which function as classic patrolling immune cells. However, murine studies reveal that tissues with high regenerative potential harbor tissue-resident eosinophils, which are highly adaptable to their microenvironment and play key roles in tissue homeostasis. This suggests the possibility that humans also possess tissue-resident eosinophils contributing to tissue homeostasis. Consequently, treatments targeting eosinophils in eosinophilic disorders should carefully consider whether they also deplete beneficial eosinophil subpopulations. It remains unclear whether eosinophilic diseases lead to the coexistence of inflammatory and homeostatic eosinophils or if pathological conditions reprogram homeostatic eosinophils into a more cytotoxic phenotype. Further research is essential to delineate these mechanisms and their implications for bone health and pathology.

## Key References

- Andreev D, Kachler K, Liu M, Chen Z, Krishnacoumar B, Ringer M, et al. Eosinophils preserve bone homeostasis by inhibiting excessive osteoclast formation and activity via eosinophil peroxidase. Nat Commun. 2024;15 [1]:1067.
  - This original study lays the foundation for the following review, as it is the first to examine the direct impact of eosinophils on bone homeostasis and pathological bone loss in both mice and humans. The manuscript highlights the interaction between eosinophils and osteoclasts, demonstrating that eosinophils inhibit osteoclast differentiation and activity through the release of eosinophil peroxidase. Mechanistically, this heme peroxidase suppresses reactive oxygen species in early osteoclasts, thereby inhibiting downstream MAPK signaling. In humans, eosinophil numbers and activity correlate with higher bone mineral density in both healthy individuals and rheumatoid arthritis patients.
- Omata Y, Frech M, Saito T, Schett G, Zaiss MM, Tanaka S. Inflammatory Arthritis and Bone Metabolism Regulated by Type 2 Innate and Adaptive Immunity. Int J Mol Sci. 2022;23 [3].
  - An excellent review summarizing current knowledge on the impact of type 2 immunity-associated cytokines on bone metabolism, particularly their role in osteoclast-mediated bone resorption.
- Zhao X, Lin S, Li H, Si S, Wang Z. Myeloperoxidase Controls Bone Turnover by Suppressing Osteoclast Differentiation Through Modulating Reactive Oxygen Species Level. J Bone Miner Res. 2021;36 [3]:591–603.
  - This manuscript demonstrates the anti-osteoclastogenic role of heme peroxidases, with a focus on myeloperoxidase. Mechanistically, myeloperoxidase inhibits osteoclasts by downregulating reactive oxygen species. Consequently, mice deficient in this enzyme exhibit reduced bone mass.
- Andreev D, Liu M, Kachler K, Llerins Perez M, Kirchner P, Kolle J, et al. Regulatory eosinophils induce the resolution of experimental arthritis and appear in remission state of human rheumatoid arthritis. Ann Rheum Dis. 2021;80 [4]:451 − 68.
  - This original study was the first to report the pro-resolving function of eosinophils in rheumatoid arthritis and to utilize single-cell RNA sequencing to map eosinophil subtypes across different tissues. Activation of eosinophils in the synovium, triggered by eosinophilic asthma, led to the resolution of inflammatory arthritis in mice. While the exact mechanism remains unclear, it involves the secretion of resolvins and the polarization of anti-inflammatory macrophages within the inflamed synovium. In humans, eosinophils were detected in the synovial tissue only in rheumatoid arthritis patients in remission.
- 60. Qin Y, Jin HZ, Li YJ, Chen Z. Emerging Role of Eosinophils in Resolution of Arthritis. Front Immunol. 2021;12:764825.
  - An excellent review summarizing current knowledge on the role of eosinophils in inflammatory arthritis.

## Data Availability

No datasets were generated or analysed during the current study.
